# Transcriptomic Data Sets for Zymomonas mobilis 2032 during Fermentation of Ammonia Fiber Expansion (AFEX)-Pretreated Corn Stover and Switchgrass Hydrolysates

**DOI:** 10.1128/mra.00564-22

**Published:** 2022-08-22

**Authors:** Yaoping Zhang, Kevin S. Myers, Michael Place, Jose Serate, Dan Xie, Edward Pohlmann, Alex La Reau, Robert Landick, Trey K. Sato

**Affiliations:** a DOE Great Lakes Bioenergy Research Center, University of Wisconsin-Madison, Madison, Wisconsin, USA; University of Delaware

## Abstract

The transcriptomes of Zymomonas mobilis 2032 were captured during the fermentation of ammonia fiber expansion (AFEX)-pretreated corn stover and switchgrass hydrolysates containing different concentrations of glucose and xylose. RNA samples were collected when Z. mobilis was fermenting glucose or xylose. Here, we present transcriptome sequencing (RNA-Seq) data obtained during separate phases of glucose or xylose consumption.

## ANNOUNCEMENT

Zymomonas mobilis is a natural ethanologen with a number of traits shared by other well-known industrial biofuel producers. Z. mobilis 2032 is a derivative of the model strain ZM4 with multiple directed and evolutionary engineering modifications that enable diauxic catabolism of xylose (1). Previous results indicated that the 2032 strain can ferment glucose to ethanol in both 6% and 9% glucan loading ammonia fiber expansion (AFEX)-pretreated corn stover hydrolysate (ACSH) and 7% glucan loading AFEX-pretreated switchgrass hydrolysate (ASGH) (1, 2). Compared to fermentation of 6% ACSH or 7% ASGH, Z. mobilis 2032 grew more slowly in 9% ACSH, consuming little xylose after glucose depletion (3). This inhibition of xylose utilization is probably caused by osmotic stress responses and/or higher concentrations of inhibitors in 9% ACSH. To better understand the physiological responses of Z. mobilis during diauxic fermentation of glucose and xylose in the different hydrolysates, we collected Z. mobilis 2032 samples at time points at which glucose or xylose was being primarily consumed and sequenced the RNA for transcriptome analyses.

**FIG 1:**
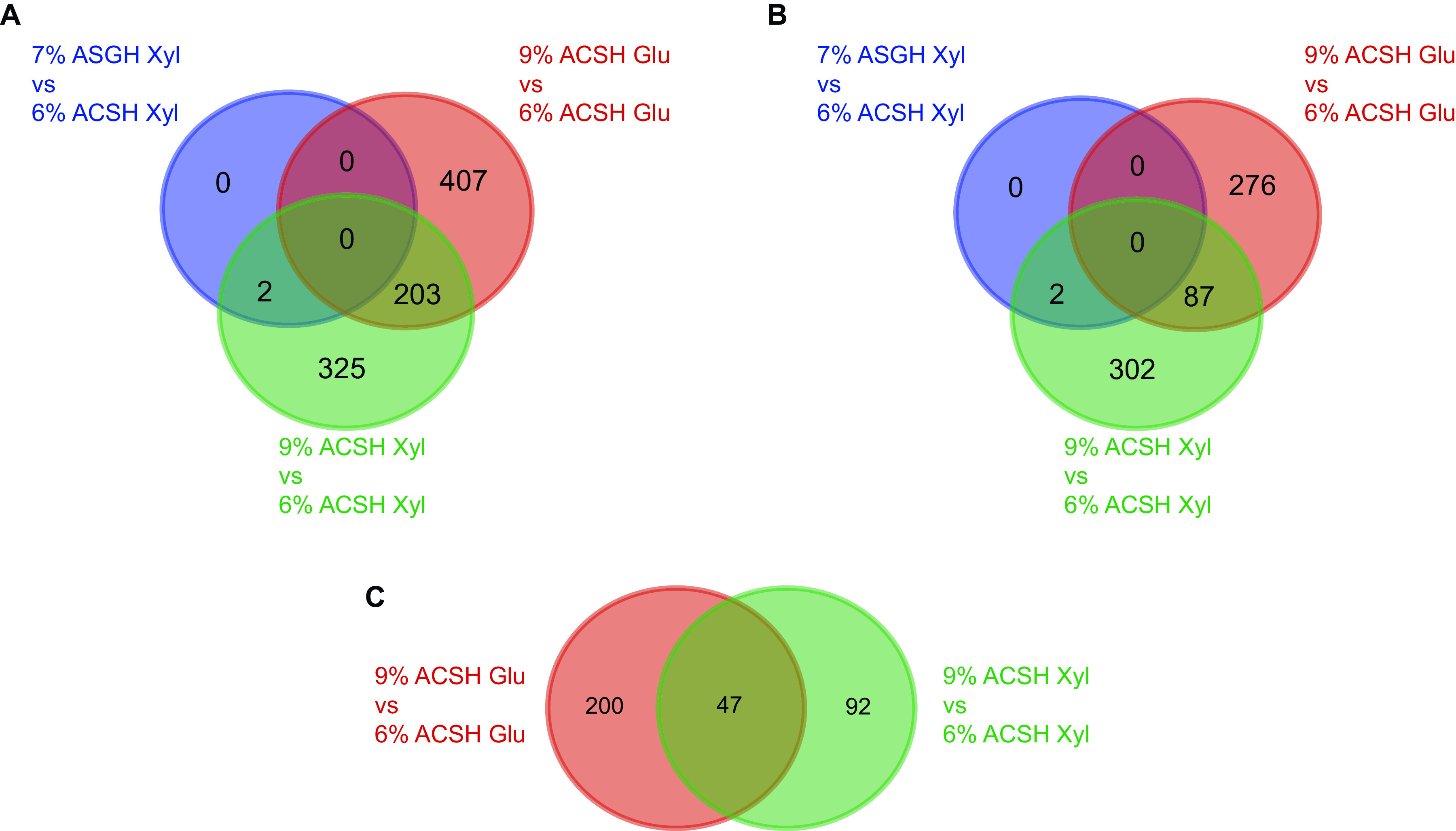
Summary of genes with significant DE by RNA-Seq under different growth conditions. (A) All genes with significant DE. (B) Genes with a significant decrease in expression, relative to the corresponding 6% ACSH growth condition. (C) Genes with a significant increase in expression, relative to the corresponding 6% ACSH growth condition. Xyl indicates the xylose consumption phase, while Glu indicates the glucose consumption phase. Note that 7% ASGH glucose versus 6% ACSH glucose had no genes with significant DE, and there were no genes with significant DE with an increase in expression when 7% ASGH xylose was compared to 6% ACSH xylose. Venn diagram circles are not proportional, to show the lack of overlap.

Fermentation of Z. mobilis 2032 was conducted in 6% and 9% ACSH and in 7% ASGH in bioreactors as described previously (1). Glucose and xylose concentrations were monitored with a YSI 2700 Biochemistry Analyzer (YSI, Inc., Yellow Springs, OH) during the fermentation. For transcriptome analyses, cells were collected at time points at which glucose or xylose was being primarily consumed, and RNA was isolated and treated with DNase as described previously (4), with at least three biological replicates (Table 1). RNA samples were delivered to the Joint Genome Institute (JGI) (Berkeley, CA) for library preparation and sequencing. After rRNA removal via the Ribo-Zero rRNA removal kit (Illumina, San Diego, CA) and paired-end library generation using the Illumina TruSeq stranded mRNA preparation kit, the libraries were sequenced with 100-bp paired-end reads on an Illumina HiSeq 2000 system. Reads were trimmed using Trimmomatic (version 0.39) (5) and were mapped to the Z. mobilis 2032 genome (GenBank accession number CP023677) using bwa-mem (version 0.7.17-h5bf99c6_8) (6) with default parameters. Alignment files were cleaned and sorted with Picard tools (version 2.26.10) (https://broadinstitute.github.io/picard) and SAMtools (version 1.2) (7). Aligned reads were mapped to gene boundaries using HTSeq (version 0.6.0) (8) and normalized using reads per kilobase per million mapped reads (RPKM). Significant differential expression (DE) was determined to be at least a 2-fold change in gene expression with an edgeR pairwise-analysis false-discovery rate (FDR) of ≤0.05 (9). There were no genes with significant DE when 7% ASGH and 6% ACSH were compared during the glucose consumption phases and only 2 genes with significant DE during the xylose consumption phases (Fig. 1). A total of 610 and 530 genes with significant DE were identified in comparisons of 9% and 6% ACSH during the glucose and xylose consumption phases, respectively (Fig. 1). The small overlap of the genes with significant DE in the different phases suggests unique responses to each hydrolysate condition.

**TABLE 1:** Summary of RNA-Seq sample data

Sample	Growth medium	Sugar consumption phase	Replicate no.	BioSample accession no.
Glu_6_A	6% ACSH	Glucose	1	GSM6053149
Glu_6_B	6% ACSH	Glucose	2	GSM6053150
Glu_6_C	6% ACSH	Glucose	3	GSM6053151
Glu_6_D	6% ACSH	Glucose	4	GSM6053152
Glu_6_E	6% ACSH	Glucose	5	GSM6053153
Xyl_6_A	6% ACSH	Xylose	1	GSM6053154
Xyl_6_B	6% ACSH	Xylose	2	GSM6053155
Xyl_6_C	6% ACSH	Xylose	3	GSM6053156
Xyl_6_D	6% ACSH	Xylose	4	GSM6053157
Glu_7_A	7% ASGH	Glucose	1	GSM6053158
Glu_7_B	7% ASGH	Glucose	2	GSM6053159
Glu_7_C	7% ASGH	Glucose	3	GSM6053160
Xyl_7_A	7% ASGH	Xylose	1	GSM6053161
Xyl_7_B	7% ASGH	Xylose	2	GSM6053162
Xyl_7_C	7% ASGH	Xylose	3	GSM6053163
Glu_9_A	9% ACSH	Glucose	1	GSM6053164
Glu_9_B	9% ACSH	Glucose	2	GSM6053165
Glu_9_C	9% ACSH	Glucose	3	GSM6053166
Glu_9_D	9% ACSH	Glucose	4	GSM6053167
Glu_9_E	9% ACSH	Glucose	5	GSM6053168
Xyl_9_A	9% ACSH	Xylose	1	GSM6053169
Xyl_9_B	9% ACSH	Xylose	2	GSM6053170
Xyl_9_C	9% ACSH	Xylose	3	GSM6053171
Xyl_9_D	9% ACSH	Xylose	4	GSM6053172
Xyl_9_E	9% ACSH	Xylose	5	GSM6053173

These transcriptomic data sets will enable investigators to identify the physiological responses to lignocellulose conversion that impact the production of sustainable biofuels by Z. mobilis.

### Data availability.

Raw RNA-Seq reads are available at NCBI GEO under accession number GSE201229.
